# How does career-related parental support benefit career adaptability of medical imaging technology students in Asia-Pacific LMICs? The roles of psychological capital and career values

**DOI:** 10.3389/fpsyg.2025.1508926

**Published:** 2025-01-24

**Authors:** Xiaohui Zhang, Yangjing Shen, Junxiang Xu, Guanyu Cui

**Affiliations:** ^1^Henan Medical College, Zhengzhou, China; ^2^College of Education, Wenzhou University, Wenzhou, China; ^3^China Youth Dream Edu-tech (Beijing) Co., Ltd, Beijing, China

**Keywords:** career adaptability, career-related parental support, psychological capital, career values, in Asia-Pacific LMICs

## Abstract

**Background:**

China, as a low- and middle-income country (LMIC) in the Asia-Pacific region, is advancing its rural medical and health system. Students of Medical Imaging Technology (MIT) in China, who will probably be employed in rural, are facing the pressure from both study and employment. Previous results proposed that career-related parent support and psychological capital could positively influence the level of career adaptability, while career-related parent support affects career values. This study aimed to explore the impact of career-related parental support on career adaptability and the mechanism between them, as well as the role of psychological capital and career values.

**Methods:**

A total of 520 (80.8% female) participants were recruited from MIT students in China, using Questionnaires about career-related parental support, psychological capital, career values, career adaptability. Path analysis was conducted using Mplus 8.3.

**Results:**

There was a significant correlation between career adaptability and career-related parental support, psychological capital and career values. Career-related parental support could positively predict students’ career adaptation. Psychological capital and career values played a parallel mediating role between career-related parental support and career adaptation.

**Conclusion:**

The results demonstrate that it is necessary to enhance career-related parental support to improve MIT students’ career adaptability by the enrichment of psychological capital and the refinement of career values, on the basis of the popularization of medical career programs among the public and medical literacy courses in colleges.

## 1 Introduction

It was reported that Doctor-to-population ratios in Asia-Pacific LMICs are well below the benchmark of World Health Organization (WHO), which is 1.15-to-1000 population (King et al., 2016). And due to the constraints of rural conditions, fewer medical graduates prefer to practice in rural areas (Chuenkongkaew et al., 2016). In order to improve the supply and retention of qualified health professionals in rural areas, ECHO programs were invested heavily aiming at providing medical students for rural areas by governments and medical colleges (Agley et al., 2021). It is no coincidence that China is boosting vocational education for developing the grass-roots, rural areas in particular (General Office of the CPC Central Committee and General Office of the State Council, n.d.). Especially, the 3-year medical program in China is mainly set up for primary medical services in rural communities, via a lower entry threshold and shorter length of schooling (Tie et al., 2024). Inevitably, these students have many more problems in career development, such as lower career adaptability (Chuang et al., 2022), more employment discrimination in job search (Liu et al., 2022). But employ-ability in curriculum was largely focused on professional skills and capabilities for a career, with gaps in research on instructional strategies to promote career adaptability (Leadbeatter et al., 2023). And literatures about career adaptability mostly concentrate on students from general universities (Guan et al., 2016). Evidently, some curriculum configurations are associated with students’ career choices (Thomas et al., 2024). There is urgent need to investigate mechanism for improving career adaptability of vocational medical college students, so as to give implications for medical school programs, thus increasing such students’ likelihood of dedicating in rural areas.

Savickas first put forward “career adaptability,” which means the attitude, ability and behavior needed by individuals to adapt to their work roles (Savickas and Porfeli, 2012). It can also be used to cope with career changes, new tasks and work setbacks (Ryan and Deci, 2017), thus in this article, career adaptability is defined as “individuals’ adjustment of their career plans facing uncertain events.” Obviously, it is an important psychological resource in times with high career mobility. Many cross-sectional studies have found career adaptability associated with positive career development (Duffy et al., 2015). People with higher adaptability showed more career planning, exploration, and self-efficacy (Boo et al., 2021). Factors contributing to students’ career adaptability mainly include demographics, proactive personality, and social support factors (such as families, peers, and significant others, etc.) (Zhang and Wang, 2019).

Significantly, parents are the most important influential factor for children’s education and career transition (Tynkkynen et al., 2010). Parents can influence children’s career self-efficacy through subtle interactions, conscious emotional support, practical help, or verbal encouragement and affirmation (Turner and Lapan, 2002). Many studies showed that career-related support from parents would enhances children’s confidence when making career development decision (Hlao and Hlouková, 2019). And parental supports can promote children’s career maturity, career exploration, and career expectation (Alfanto et al., 2019). So career-related support can be conducive to children’s career adaptability (Guan et al., 2016).

Psychological capital, a personal trait resource (Luthans et al., 2007), which could strengthen one’s confidence and ability to actively cope with demands and pressures (Calvo and García, 2021). So, high psychological capital will lead to less academic stress and burnout (Vanno et al., 2014), more academic outcomes (Datu and Valdez, 2016) and better academic performance (Datu et al., 2018; Martínez et al., 2021). Also, it can bring benefit to job performance (Luthans et al., 2010), critical thinking and personal adaptability (Calvo and García, 2021). In short, psychological capital, as a positive trait, played an important role in influencing the performance of academic and career. Psychological Capital is also proposed a mediator, for example, for the instructor support influence on postgraduate students’ well-being (Pan et al., 2018), and for family support influence on children’ well-being (Wang et al., 2022). Hence, it is reasonable that outer factors influence psychological capital and psychological capital influences behavior results.

Career values were the basis for individuals to judge the importance of career outcomes (Elizur, 1984), so it is the basis for self-evaluation and evaluation of others, influencing one’s career choice and effort paid. Thus, Career values own the nature of evaluating and orienting one’s behaviors and goals according to what is desirable (Brown, 2002). Career values will determine one’s choosing in the transition from school to work (Porfeli, 2007). Still, some research showed that different career values lead to job and life satisfaction, and thus career success (Alzyoud, 2017). While other literatures underlined the prominent effect of parental support on children’s career values directly or indirectly (Kulik, 2002) especially in collectivist societies (Sawitri et al., 2014).

Previous studies above revealed that career adaptability is helpful for career stability, especially under harsh working conditions, such as rural in Asia-Pacific LMICs. Under the background of vigorously developing rural medical conditions in China, it is very important to do research in promoting career adaptability of vocational medical college students. Since the relevant factors mentioned above may generally act dependently (Feng et al., 2021), what are the impact of career-related parental support on career adaptability and the mechanism between them, as well as the role of psychological capital and career values? Taking MIT students as an example, this study will probe into the correlation between personal and environmental factors for the purpose of finding out the key factors affecting career adaptability, and put forward some suggestions for medical education.

**Figure 1:**
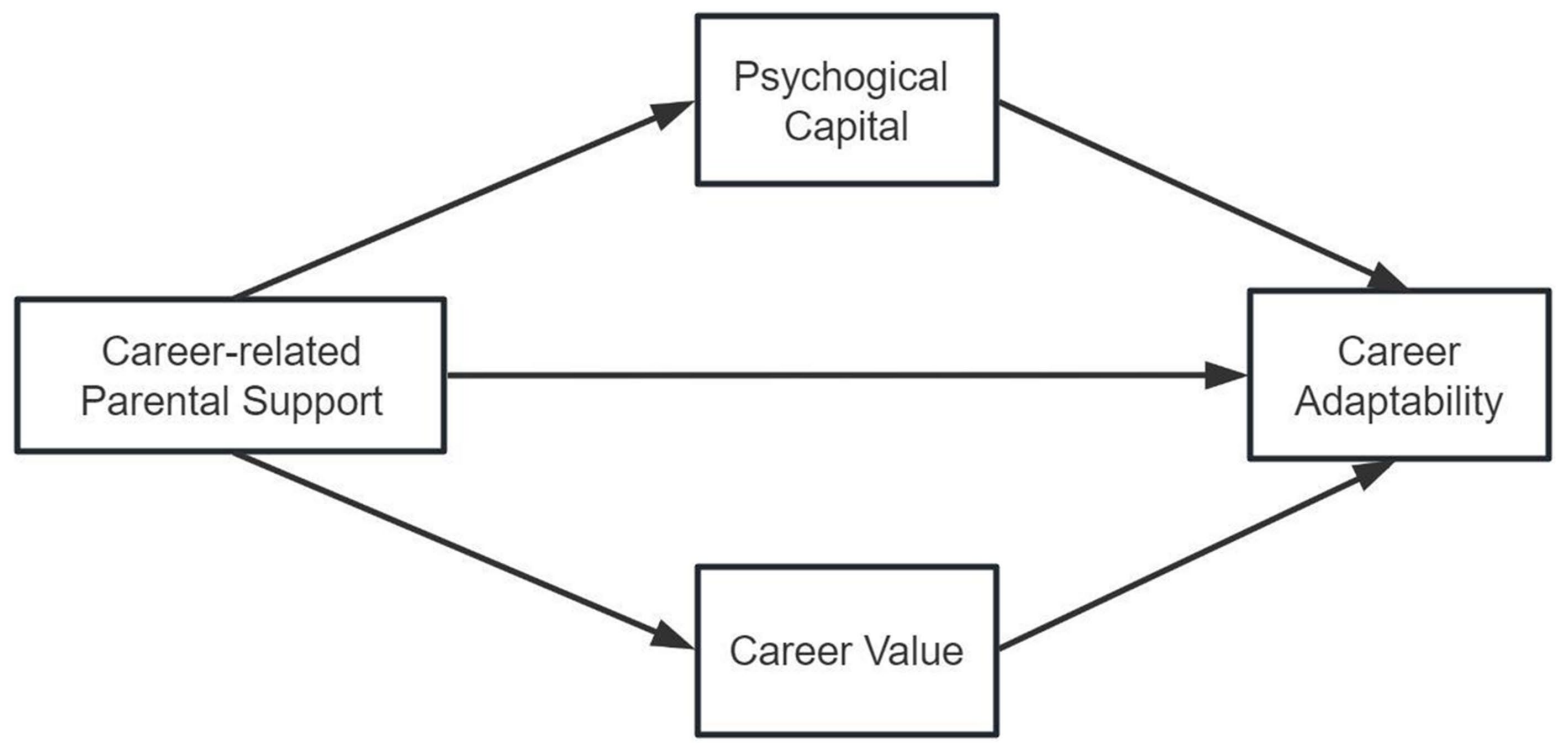
The model graph of parental support on career adaptability of vocational college students and the mediating role of psychological capital and career values.

In this study, a multiple mediation model is constructed and tested. Specifically, we examine the relationship between career-related parental support and career adaptability by examining the mediating role of psychological capital and career values (Figure 1). Based on the expansion of previous studies, we put forward the following three hypotheses:

Hypothesis 1: Career-related parental support predicts the level of career adaptability positively.

Hypothesis 2: Psychological capital plays a mediating role in the impact of career related parent support on career adaptability.

Hypothesis 3: Career values mediate career-related parental support on career adaptability.

## 2 Methods

### 2.1 Participants and procedure

A cross-sectional design was employed in this study. Consequently a cluster sampling method was adopted to distribute questionnaires in a higher vocational medical college in Henan Province, China, and questionnaires were distributed and collected by using Solump (an online questionnaire survey platform). QR code of questionnaire survey was distributed to all classes of the MIT students in this college and researchers informed them to participate this survey of their own free will. In order to ensure informed consent, detailed rules of informed consent are introduced at the beginning of the questionnaire, and the questionnaire survey can only be conducted with the approval of the subjects. After deleting the cases who answered the questionnaire less than 180 s and who answered exactly the same option, finally a total of 520 (80.8% female) colleague students were collected. The characteristics of the sample are as shown in Table 1.

**Table 1:** Characteristics of the sample (*N* = 520).

Variable		*N*	PCT	Variable		*N*	PCT
Gender	Male	100	19.2	Mother’s occupation	Work in system	31	6.0
	Female	420	80.8		individual Enterprise	89	17.1
Grade	Freshman	181	36.1		flexible employment	400	76.9
	Second	207	39.8	Father’s occupation	Work in system	46	8.8
	Junior	132	25.4		individual Enterprise	101	19.4
Home area	County and urban areas	175	33.7		Flexible employment	373	71.7
	Rustic town	345	66.3				

### 2.2 Measures

#### 2.2.1 Career-related parental support

The Career-specific parental behaviors scale developed by Dietrich and Krackewas adopted (Dietrich and Kracke, 2009), which consisted of three subscales: Parental occupational support, parental occupational involvement, and parental occupational lack of involvement, each of which had 5 items. Examples of parental career support items include: “My parents communicate with me about my career interests and abilities.” Example items of parental career intervention such as: “My parents have their own views on my future career and are trying to influence me.” Example items of parental career lack of involvement such as: “My parents cannot help me prepare for my career because they are too busy.” All the items are rated on a 5-point Likerttype scale ranging from 1 (absolutely disagree) to 5 (absolutely agree).

#### 2.2.2 Psychological capital

The 12-item Psychological Capital Questionnaire (PCQ-12) was adopted to measure psychological capital (Luthans et al., 2007). The PCQ contains four subscales: self efficacy (3 items; e.g., “I feel confident presenting information to a group of classmates”), optimism (2 items; e.g., “I am optimistic about what will happen to me in the future as it pertains to academic work”), hope (4 items; e.g., “I can think of many ways to reach my current learning goals”), and resilience (3 items; e.g., “I can get through difficult times at academic work because I have experienced difficulty before”). Participants responded to these items on a 6-point Likert scale ranging from 1 (complete conformity) to 6(complete non-conformity). Positive scoring was adopted in the questionnaire, and the higher the score, the stronger the positive psychological capital. The Cronbach’s *α* coefficient of this questionnaire in the study was measured as 0.814. The PCQ-12 has shown good internal reliability in students (Wang et al., 2022).

#### 2.2.3 Career values

Considering that the differences between Chinese and Western cultures lead to different professional values, the questionnaire prepared by Ling WS in 1999 was used in this study (Ling et al., 1999). The questionnaire included three dimensions: prestige status factors (9 items; e.g., It is easy to become famous and to be an expert.), health factors (6 items; e.g., the career is in line with my own interest.), and self-development factors (7 items; e.g., the career provide equal opportunities, fair competition). The questionnaire uses Likert 5 point scoring method to divide each professional values item into five grades: “very important, important, somewhat important, general, and not very important.” The higher the score of a dimension, the stronger the individual’s need for a value. The Cronbach’s *α* coefficient of this questionnaire in the study was 0.843.

#### 2.2.4 Career adaptability

The career adaptability of the subjects was measured by the Career Adapt-Abilities Scale (CAAS) developed by Savickas and Porfeli (2012). The scale consists of four dimensions, namely, career concern, career control, career curiosity and career confidence, including four reverse scoring questions, with a total of 24 questions. Sample items are as follows: concern (e.g., “Planning how to achieve my goals”), control (e.g., “Taking responsibility for my actions”), curiosity (e.g., “Becoming curious about new opportunities”), and confidence (e.g., “Solving problems”).

Likert 5 points were used in the scale from 1, strongly disagree, to 5, strongly agree. And the higher the score of the subjects, the stronger their adaptability in their career. The Cronbach’s α coefficient of this questionnaire in the study was 0.820.

### 2.3 Data analysis

SPSS 25.0 and Mplus 8.3 were used for data analysis. We screened the questionnaire and eliminated 46 invalid data samples before analyzing the data. Firstly, a preliminary descriptive statistical analysis and correlation analysis were conducted on the data with SPSS 25.0 to explore the correlation among career-related parental support, career adaptability, psychological capital and career values.

Then, we used the ML Estimator to estimate the parameters of the Structural Equation Model (SEM) in process to perform the mediation analysis. Repeat sampling was performed with BC Bootstrap, and the test was conducted by estimating the 90% confidence intervals of the mediating and moderating effects through 2000 samples. If the confidence intervals did not contain zero, statistical significance was indicated. Afterwards, the mediating role of psychological capital and career values between career-related parental support and career adaptability was confirmed. Ultimately, we reported the model fit indices assessed by the conventional levels of the goodness of fit. The evaluation indicators selected in this study were the comparative fit index (CFI), the Tucker–Lewis index (TLI), the standardization root mean square residual (SRMR). When CFI and TLI were greater than 0.90 and RMSEA were less than 0.08, the model was considered to fit well.

## 3 Results

### 3.1 Common method bias test

The four questionnaires used in this study were self-rated, so for the accuracy of the results, Harman common method bias test was performed on all the data before further analysis. The results showed that there were 11 factors whose characteristic root was greater than 1, among which the variance explained by the first factor was 21.996, accounting for 30.550% of the cumulative variance, which was less than 40% of the critical value, indicating that this study was not significantly affected by the common method bias.

### 3.2 Preliminary analysis

After controlling for the influence of gender and grade, we generated the fitting indices of the model: CFI = 1.00, TLI = 1.00, χ2/df = 116.73, RMSEA (90%CI) = 0.000 [0.000, 0.000]. These model fitting indexes showed this model fits well.

Table presents a correlation analysis of research variables: career-related parental support, psychological capital, career values, and career adaptability (*n* = 520). All of these research variables showed significant correlations. To be more specific, career-related parental support was positively related to career adaptability (*r* = 0.43, *p* < 0.001), psychological capital (*r* = 0.50, *p* < 0.001), and career values (*r* = 0.34, *p* < 0.001). Additionally, career adaptability was also positively related to psychological capital (*r* = 0.72, *p* < 0.001) and career values (*r* = 0.28, *p* < 0.001). Furthermore, there is also a significant positive correlation between psychological capital and career values (*r* = 0.28, *p* < 0.001) (Table 2).

**Table 2:** Correlation analysis of variables (*N* = 520).

Variable		*M*	SD	1	2	3	4
1	Career-related parental support	3.79	0.62	—			
2	Psychological Capital	4.53	0.71	0.50***	—		
3	Career Values	3.70	0.50	0.34***	0.28***	—	
4	Career Adaptability	3.48	0.41	0.43***	0.72***	0.28***	—

Gender was dummy coded as 1 = male and 2 = female. *p* > 0.05, **p < 0.01, ***p < 0.001.

### 3.3 Hypothesis testing

**Figure 2:**
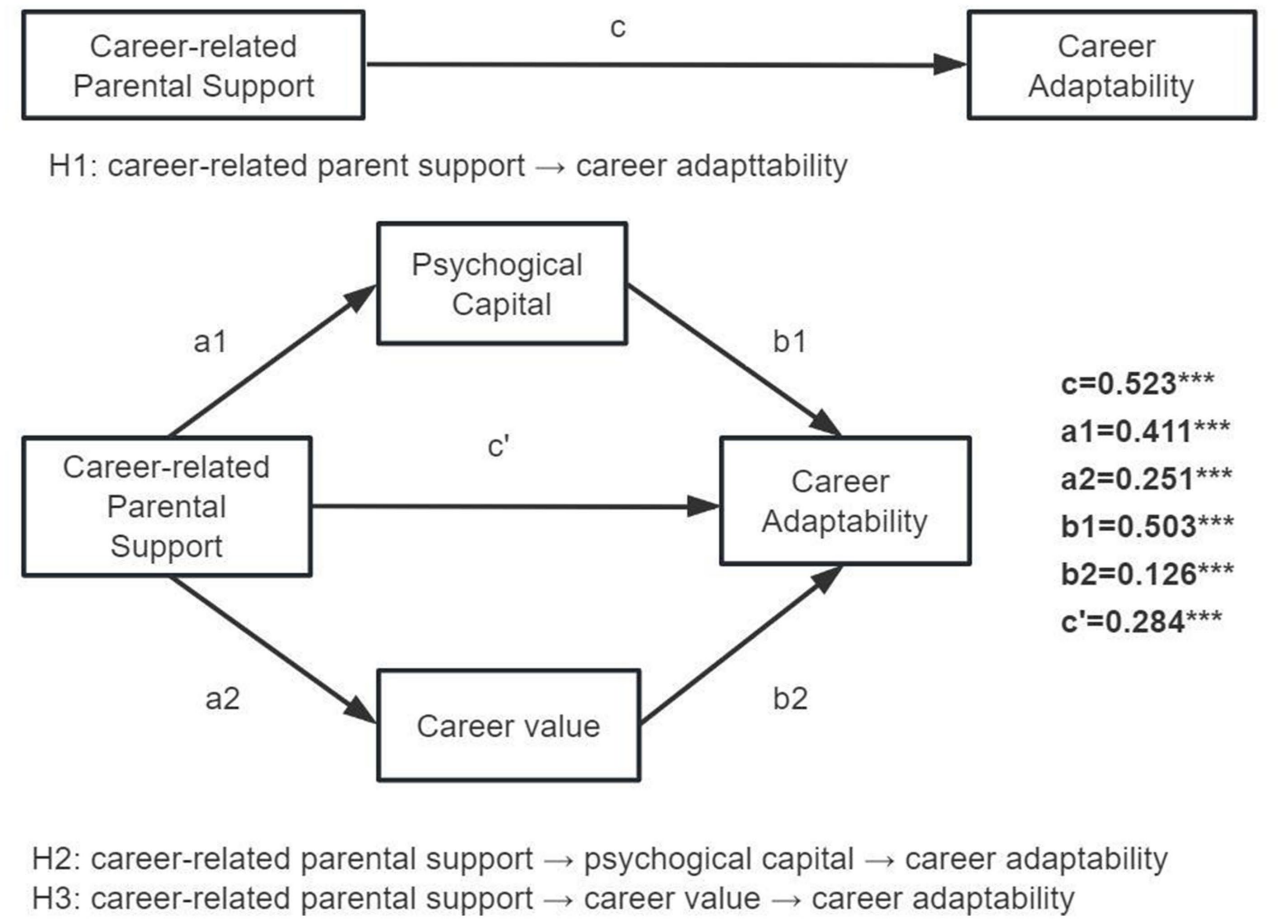
In this model, the indent effect of career-related parental support on career adaptability through psychological capital. *N* = 520; *p* > 0.05, * *p* < 0.05, *** *p* < 0.001.

First of all, before adding other variables, we examined the predictive role of career-related parental support on career adaptability through the path analysis procedure in Mplus (Figure 2). The results showed that career-related parental support had a positive predictive effect on career adaptability (*β* = 0.523, *p* < 0.001), verifying Hypothesis 1.

Secondly, in order to test Hypotheses 2 and 3, psychological capital and career values were added into the model (see Figure 2). The results showed that career-related parental support could significantly predict psychological capital (*β* = 0.411, *p* < 0.001) and career adaptability (*β* = 0.284, *p* = 0.042); psychological capital significantly predict career adaptability (*β* = 0.503, *p* < 0.001). Moreover, career-related parental support significantly predicts career values (*β* = 0.251, *p* < 0.001); career values significantly predict career adaptability (*β* = 0.126, *p* < 0.001). In addition, as shown in Table, the profile effect evaluation of this model shows that psychological capital (90% CI = [0.193, 0.312]) and career values (90% CI = [0.017, 0.070]) have two independent mediating effects between career-related parental support and career adaptability (Table 3).

**Table 3:** Bootstrap mediation effect table (*N* = 520).

	Point estimate	Product of confidence		BOOTSTRAP 2000 TIMES 90%CI	
		S.E.	Est/S.E.	Lower	Upper
Direct effect					
Career-related parental support → career adaptability	0.346***	0.039	8.913	0.268	0.423
Indirect effect					
Career-related parental support → psychological capital → career adaptability	0.251***	0.030	8.285	0.193	0.312
Career-related parental support → career values → career adaptability	0.039**	0.013	2.902	0.017	0.070

*p* > 0.05, **p < 0.01, ***p < 0.001.

## 4 Discussion

In China, vocational medical education serves as the cornerstone of healthcare systems in rural communities, with the lower college admission score. So, most students are recommended to choose medical majors by parents without any career planning. Then once in colleges, with the gradual understanding of the major and corresponding career in rural and remote, students will carry on their career choice again. Furthermore, with the rapid development of society, students are faced with great career uncertainty, and frequent career changes have become a social phenomenon that cannot be ignored. According to the existing research results, career adaptability plays an even more important role in such unpredictable times (Hirschi and Valero, 2015). In order to help these students to clear major goals, stable learning determination, and improve future employ-ability, it’s better to propose suggestions for teaching reform of medical vocational education. As a result, they can help these students to utilize resources fully to adapt to the rural medical career and serve local residents more efficiently. And eventually these findings and recommendations can provide lessons for other LMIC how to facilitate healthcare providers to sink into grassroots rural areas.

### 4.1 Direct effect of career-related parental support on career adaptability

Many previous studies have suggested that parents will have an important influence on children in the stage of career exploration (Duffy et al., 2015; Zhang and Wang, 2019). In the process of our research, we further verified the positive predictive effect of career-related parental support on students’ career adaptability with data analysis, which coincides with the hypothesis proposed above. Career-related parental support, including career-related advice and guidance to young people, when necessary (Guan et al., 2015), can contribute significantly to a student’s career adjustment. To a certain extent, the more support students receive from their parents, the faster and better they can adapt to their professional roles directly, so we recommend that parents provide emotional or technical support to their children when conditions permit. This support, whether expressed through words or actions, is very helpful to the child’s career adaptation. At the same time, we also consider whether there are other operable variables in this path that can help improve students’ career adaptability. Therefore, we delve into the role of psychological capital and professional values, which we discuss in detail below.

### 4.2 Mediating roles of psychological capital and career values

Data analysis shows that career-related parental support is a significant predictor of psychological capital, psychological capital has a positive impact on career adaptability, and psychological capital plays an intermediary role between career-related parental support and career adaptability, verifying hypothesis 2. Career-related parental support can improve the psychological capital of individuals. The more support individuals feel, the higher their use of support, which also leads to the higher level of psychological capital. Individuals with better psychological capital show a higher desire for success, which will also make them face difficulties and challenges with a more positive attitude, improve the determination to overcome difficulties, and raise the probability of success (Calvo and García, 2021). Good self-efficacy makes people full of confidence in success, full of hope can enhance people’s will and explore the road to success, optimism makes people realistic and flexible, and flexibility can make people recover and surpass themselves as soon as possible (Chuang et al., 2022). In the current situation, concordant interactive within the family could decrease individuals’ perceived stress, thereby improving self-efficacy, enhancing expectations for the future, coping with challenges optimistically and positively, and improving psychological capital (Poots and Cassidy, 2020). In addition, due to the failure of the college entrance examination, 3-year medical students choose colleges and majors passively. The orientation of future study and employment is not clear, resulting in a low level of psychological capital, so they may have strong learning motivation, but weak willpower; social orientation is more rational, but confidence in job prospects is not strong. So vocational colleges, an important force for ensuring social accountability and sustainability, should not only prepare learners just for practice, but to become future change agents (Dubé, 2024). The cognitive courses about psychological adjustment and career planning will consolidate motivation (Diwan et al., 2013), strengthen academic orientation and career goal orientation (An et al., 2023). So, intervention programs in literacy courses, such as carrying out career planning consultation, organizing simulated job fairs and other activities will help students fully adapt to the social needs and future challenges. In other words, psycho-social skills training helps improve their skill and well-being (Malins et al., 2023), to actively play their own advantages.

In the process of dealing with hypothesis 3, this paper confirms the mediating role of career values between career-related parental support and career adaptation. The support of career-related parents has an important impact on vocational college students’ career values. Many studies believe that parents’ attitude will have a profound impact on children’s future career goals and pursuits (Mok et al., 2021). Driven by professional values, students are more willing to work hard to develop adaptability for career development (Ye, 2020) and more actively participate in career self-management (Jackson and Tomlinson, 2019). At the same time, the positive effect of career values on career adaptability is also confirmed in this study. If students can develop their professional values scientifically and reasonably, then these values can interact smoothly with their professional characteristics, help them objectively analyze and deal with problems and contradictions, and ultimately achieve better self-development (Playford et al., 2021). Nowadays, to work in rural communities is the need and the trend for medical students (Liu and Zhang, 2019). If parents point out the value of working in rural hospitals, it will be more beneficial for students to form reasonable career values that are more conducive to their future career development. Notably, publicizing the importance of rural medical care from the social level and shaping parents’ employment view will help students choose rural healthcare.

In conclusion, students’ psychological capital and career values play a partial mediating role between career-related parental support and students’ career adaptability. If we want to make career-related parental support play a good role in career adaptability, we must pay attention to the intrinsic influence of parents on students’ self-efficacy and psychological resilience. At the same time, it is a must to attach importance to the influence of parents on children’s professional values (Brown, 2002), which is undoubtedly an important factor related to students’ career adaptability (Russo and Stoykova, 2015).

### 4.3 Strength and limitations

The findings of this study have important practical implications. As an important predictor of MIT students’ career adaptability, career-related parental support can be used as an important tool to help their career development. Therefore, when designing relevant interventions to enhance career adaptability of MIT students, researchers or educators should also pay attention to parents’ support. It is necessary to strengthen the presentation and education of career planning to the public, so as to guide parents to offer positive support for children’s career, thus to steer their career values and improve their psychological capital. In short words, only after parents understand the imperative employment of rural medical workforce in rural, could they provide effective verbal encouragement, valuable emotional support, work mode support and vocational skills support to these students, to help them employ-ability. In addition, it is worth noting psychological capital. The durability of such training effects has been examined (Yeh et al., 2023). Among medical vocational students, it is imperative to carry out such training in medical literacy courses, especially in the transitions from college to clinic (Rachoin et al., 2023), or focus on curricular design to optimize student satisfaction with career decisions (Putri et al., 2020).

With the development of economy and the improvement of people’s health awareness, the need for a greater focus on rural community health is getting greater. Just because of the scarcity of medical resources, some Asia-Pacific LMICs are devoting to some projects to promote rural medical care (Podsakoff et al., 2003). Similarly in China, vocational Medicine shoulder the responsibility of rural health care in future. For the whole society, it is necessary to publicize basic conditions of social labor supply and demand, so as to help parents form an accurate understanding of the employment of medical major students. Through parent–child interaction, students can shape an objective career values. Meanwhile, colleges shall strengthen humanistic literacy courses, thereby to improve students’ career adaptability. Findings and suggestions above, may be referred in other countries, to lead medical students to join the rural medical system.

Despite the theoretical and practical implications discussed above, our study still has the following shortcomings that need to be improved. Firstly, although the participants in our study are representative of MIT students, they were all from the same vocational undergraduate institution. From this point of view, the scope of the study can be expanded to investigate more vocational students from different colleges in future research. Secondly, the participants in this study were only higher vocational students in Medical Imaging. In the future, research can be carried out for students of different majors to expand the influencing factors of students’ career adaptability in different industries, and effective suggestions can be put forward for more vocational students, emphasizing that there is no one-size-fits-all approach (Yeh et al., 2023). Last but not least, the samples of this study were collected in a one-time manner through self-report measurements, which may affect the accuracy of the samples (Podsakoff et al., 2003). If conditions permit, appropriately increasing the data sources for other evaluations and conducting multiple measurements may be more helpful for the validity of the research results.

## 5 Conclusion

The results of this study expand the existing research on medical education in Asia-Pacific LMICs from the perspective of career adaptability of MIT students. This study emphasizes the importance of career-related parental support in positively predicting students’ career adaptability. Parents’ support in career can empower students’ psychological capital and help students adapt to career better. The influence of career-related parental support on students’ career values also plays an important role in students’ future career adaptability. Under context of the rural health care boosting, the study puts forward some suggestions of medical education, which has practical significance to accelerate the construction of modern vocational education system. Future research can improve the model on the basis of expanding the scope of research, and help put forward more comprehensive and effective recommendations for the diverse landscape of medical education in LMICs.

## Data Availability

The original contributions presented in the study are included in the article/supplementary material, further inquiries can be directed to the corresponding author.
